# 3D investigation shows walls and wall-like structures around human germinal centres, probably regulating T- and B-cell entry and exit

**DOI:** 10.1371/journal.pone.0242177

**Published:** 2020-11-10

**Authors:** Miguel Thomos, Patrick Wurzel, Sonja Scharf, Ina Koch, Martin-Leo Hansmann

**Affiliations:** 1 Reference and Consultant Center of Lymph Node and Lymphoma Pathology at Dr. Senckenberg Institute for Pathology, Goethe-Universität Frankfurt am Main, Frankfurt/Main, Hessen, Germany; 2 Department of Molecular Bioinformatics, Johann Wolfgang Goethe-University Frankfurt/Main, Frankfurt/Main, Hessen, Germany; 3 Frankfurt Institute for Advanced Studies (FIAS), Frankfurt/Main, Hessen, Germany; University of Texas Medical Branch at Galveston, UNITED STATES

## Abstract

This study deals with 3D laser investigation on the border between the human lymph node T-zone and germinal centre. Only a few T-cells specific for antigen selected B-cells are allowed to enter germinal centres. This selection process is guided by sinus structures, chemokine gradients and inherent motility of the lymphoid cells. We measured gaps and wall-like structures manually, using IMARIS, a 3D image software for analysis and interpretation of microscopy datasets. In this paper, we describe alpha-actin positive and semipermeable walls and wall-like structures that may hinder T-cells and other cell types from entering germinal centres. Some clearly defined holes or gaps probably regulate lymphoid traffic between T- and B-cell areas. In lymphadenitis, the morphology of this border structure is clearly defined. However, in case of malignant lymphoma, the wall-like structure is disrupted. This has been demonstrated exemplarily in case of angioimmunoblastic T-cell lymphoma. We revealed significant differences of lengths of the wall-like structures in angioimmunoblastic T-cell lymphoma in comparison with wall-like structures in reactive tissue slices. The alterations of morphological structures lead to abnormal and less controlled T- and B-cell distributions probably preventing the immune defence against tumour cells and infectious agents by dysregulating immune homeostasis.

## Introduction

The human immune system is decentralised and includes about 300 to 700 lymph nodes. Each lymph node drains a special peripheral area and is divided into compartments, such as a T-, as well as a B-zone. Both compartments feature different specialized networks of reticular cells, which guide the lymphocyte movement. The T-cell compartments show collagen structures, which are ensheathed by fibroblastic reticular cells forming sinuses. In contrast, the B-cell compartments show a network of follicular dendritic reticulum cells (FDC) enabling the germinal centre’s (GC) cell reactions [1, 2]. This investigation focuses on the T-cell compartments consisting of fibroblastic reticular cells (FRC), forming sinus structures. The FRC sinus network is filled with lymphoid cells of the T-zone leading to homeostasis between network structure and T-cell amount and distribution. The strongly alpha-actin positive FRCs secrete interleukin 7 to prevent naive T cells from apoptosis [3, 4]. The T-zone provides a reservoir of CD4 and CD8 cells stimulated by survival factors and interacting with antigen presenting cells (APC) and macrophages. Chemokine secretion is essential to keep and guide the respective lymphoid cells in the specific compartments [5, 6]. The T-zone is mainly controlled by CCL19, and CCL21 including CCR7 [6–8]. Lymphocytes enter the T-zone by passing high endothelial venules (HEV) supported by LFA1, L-Selectin and ICAM1/2 [9]. Additional factors like CXCL13, BCL6, ICOS and PD1 promote T-cell differentiation and germinal centre entry [10, 11]. A former 3D investigation has shown different surface-volume quotients of FRCs in LAD compared to malignant lymphoma [12]. This leads to the assumption that the delicate B-/T-border cannot maintain the cellular homeostasis due to morphologic changes in lymphoma. Furthermore, fast B-cell movements have been traced in a previous study within the bordering region between B- and T-zone [13]. Therefore, it is questionable, whether there is a leading sinus or an FRC structure promoting fast movements. The aim of this work is to examine sinus and FRC structures of the transition between T-zone and the border of the germinal centre using 3D laser scanning technology.

## Methods

### Sample preparation

Tissue blocks for this investigation were taken from the Reference and Consultant Centre of Lymph Node and Lymphoma Pathology at the University of Frankfurt/Main. No ethical concerns are raised over the use of the anonymized archived surplus tissue in the project. A waiver from a formal Institutional Review Board application has been granted. The author affiliated to the Reference and Consultant Centre of Lymph Node and Lymphoma Pathology was involved in the tissue collection.

Two different types of lymphadenopathy were investigated in 3D, the lymphadenitis (LAD) and as a neoplastic counterpart the angioimmunoblastic T-cell lymphoma (AITL). The AITL was selected as an example of a highly destructive germinal centre (GC) process. Moreover, cancer associated fibroblasts (CAF) play an important role in angiogenesis and the destruction of the connective tissue in AITL [14].

Formalin-fixed and paraffin-embedded lymph nodes were used and processed as described previously [15]. In brief: About 12–25 μm thick paraffin sections were cut and incubated. After the antigen retrieval by pressure cooking in EDTA (pH 8, 90 seconds), the sections were transferred into purified water and incubated by monoclonal antibodies. We used anti-alpha smooth muscle actin antibody (SP171/Abcam), anti-human BCL6 monoclonal mouse antibody (M7211;Agilent/Dako), anti-human polyclonal rabbit IgD antibody (IR517;Agilent/Dako), anti-human CD3 monoclonal mouse antibody (A0452; Agilent/Dako) and anti-human CD23 monoclonal mouse antibody (M7312; Agilent/Dako). Additionally, VectaFluor ready-to-use antibody Kit (Cat. No. DK-8818) (secondary Antibody) for imaging and DAPI nuclear acidic staining (D9542; Sigma-Aldrich). The staining was performed as specified by the manufacturer and was limited to 3 stainings per specimen. For good visualisation of myofibroblasts we used an anti-alpha actin antibody instead of beta-actin.

### 3D imaging

For the 3D scan a Leica TCS SP8 confocal microscope (Leica Microsystems, Wetzlar 35578 Germany) was used [15] with the objective: HC PL APO 63x/1.3 GLYC CORR Cs2, lasers: 405 nm DMOD Compact, Red 594 nm and Green 488 nm. The pixel size was set to 130 nm in x, y and z direction. The z-step size was set to 0.13.

Most of the LAD images demonstrated a germinal centre in the middle of the image stained with Anti-CD23. Some images showed parts of a germinal centre and were also included in the study. However, AITL cases showed the destruction or partial regression of germinal centres. Of special interest in these cases was the evaluation of the transition zone between T-zone and germinal centre illustrating wall-like structures at the B-/T-border. In addition, at this border region the transition zone appeared to be negative for alpha-actin and CD23.

### Image processing

Methodology according to the paper of Oswald et al. [12] and adapted to our study.

Imaris Advanced Tracking 9.2 from Bitplane AG (Badenerstraße 682, CH-8048 Zurich, Switzerland) was used for image processing. To examine the FRC wall structures around the germinal centres, the perifollicular region has been set as a Region Of Interest (ROI) around every GC, i.e. the whole perifollicular region. In case of images showing more than one germinal centre, all the germinal centres were examined and selected as a ROI within a huge rectangular selection field. In AITL, where germinal centres could not clearly be identified, the entire image was selected as ROI. After the ROI selection the surface of the immune-stained structures were reconstructed by Imaris using the “automatic surface reconstruction” and therefore resulting in comparable surfaces for different cases. Exploring different images, a threshold was chosen depending on the intensity of fluorescence activity. For automatic determination of thresholds, the software uses the “k-means algorithm” [16]. If the fluorescence staining of the tissue was not intense enough, threshold determination was adjusted manually till it was comparable to the other cases. Finally, the surface was generated by the “marching cubes algorithm” [17]. Then, the selected ROI showed the calculated surface. In order to avoid auto-fluorescence and cell fragments from different planes of the tissue, surface parts which are smaller than 35 μm^3^ were filtered and removed. The filtering procedure clearly enhanced the image quality.

We identified the FRC walls and wall-like structures by morphological criteria, following generating the 3D surface. The structures were located around the germinal centre on the B-/T-border, showing a consistent height and length, which demonstrated a smooth surface. The orientation of these walls was within a plane of the examined layer’s thickness and defined a clear boundary between the two compartments. Blood vessels were also visualized by the alpha-actin antibody but not further evaluated in this investigation.

### Data acquisition

We proceeded with the calculated surfaces and used the Imaris feature “Measurement Points” to obtain data about the length of the wall and wall-like structures, wall thickness, wall height in the z-dimension, gaps within these walls, as well as the width of perifollicular sinuses. The data were exported for calculations and statistics with the “stats packet” of the Python 3.6.1. library SciPy [18]. In doing so, we used the Kolmogorov-Smirnov Test to calculate for equality of the given distributions [19].

## Results

### Description of 3D representable FRC walls

**Fig 1:**
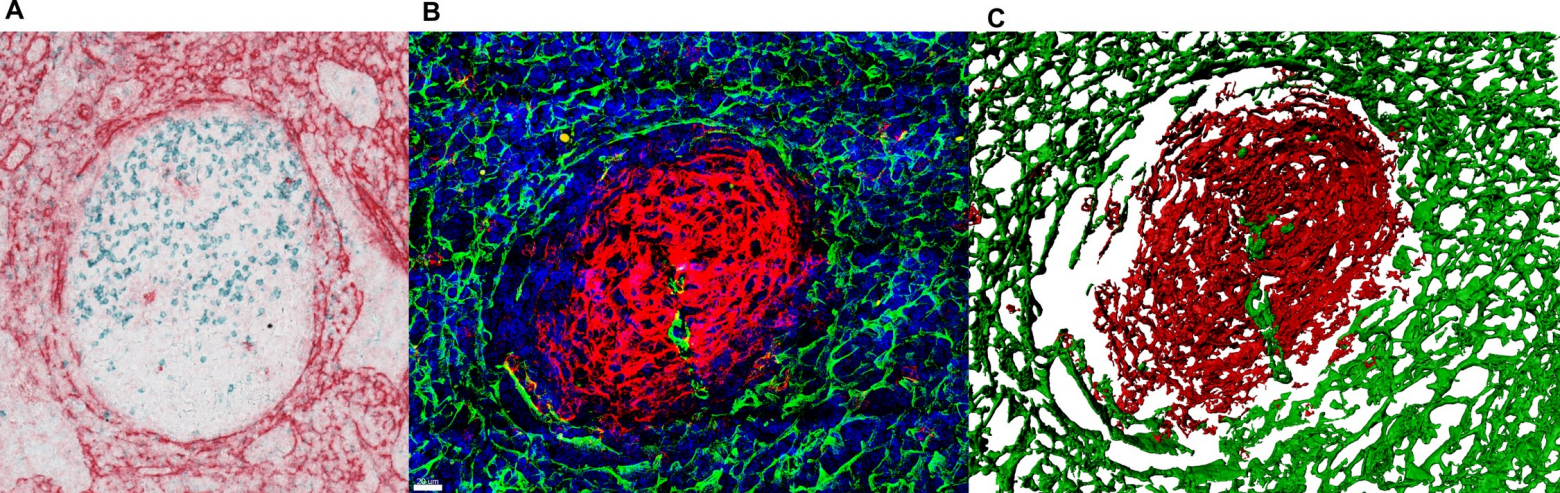
Image quality and information enhancement from 2D to 3D. (A) PD1^+^ (blue) and alpha-actin^+^ (red) cells in standard 2D specimen. The germinal centre is situated in the middle of the picture, surrounded by the T-zone. (B) Confocal microscope image shows follicular dendritic cells (FDC) in red (CD23^+^) and fibroblastic reticular cells (FRC) in green (alpha-actin^+^). (C) Constructed surfaces for the confocal microscopy image in Image 2 (FDC in red and FRC in green). This surface reconstruction illustrates the three-dimensionality of the tissue. As can be seen, the walls in B-/T-border are alpha-actin positive structures that can be related with the FRCs. Magnification of microscopy images. (A) 40x (B and C) 63x.

**Fig 2:**
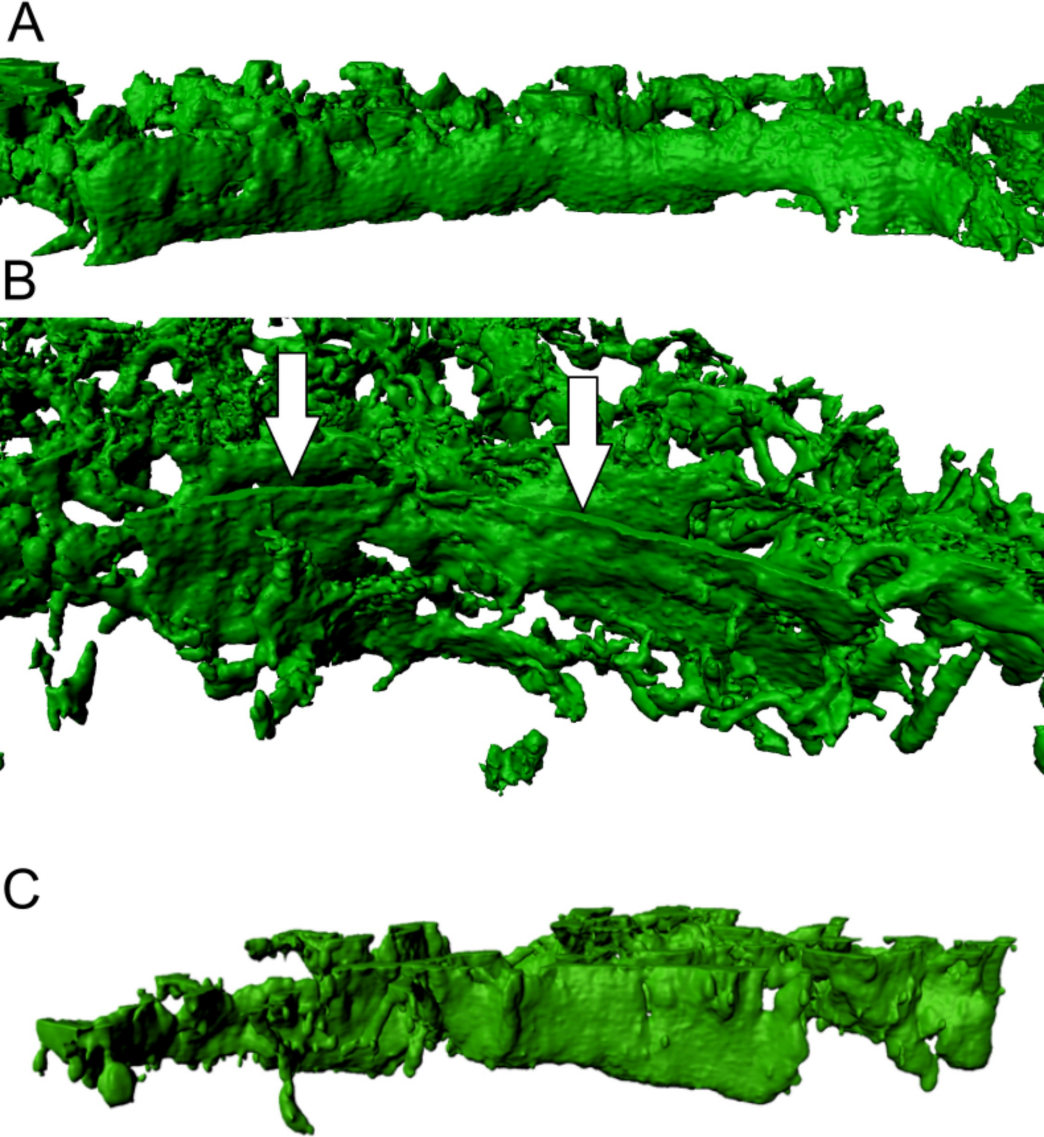
Morphological appearance of alpha-actin positive fibroblastic reticular cell (FRC) walls in lymphadenitis (LAD). (A) 3D view from the germinal centre towards the T-zone shows clear B/T-border compartmentalisation by FRC walls in lymphadenitis. This image contains a consistent FRC wall defining a clear barrier between two lymph node compartments. Arrows (in white) point on an FRC wall. (B) The point of view of this image is from the germinal centre towards the bordering T-zone, depicting a FRC wall. (C) FRC wall with a clearly defined border towards B-zone. The surface is smoother and more regular in comparison to the alveolate structure within the T-zone. The maximum resolution of (A), (B) and (C) was set to 0.13 μm per pixel in the native microscopy dataset.

**Fig 3:**
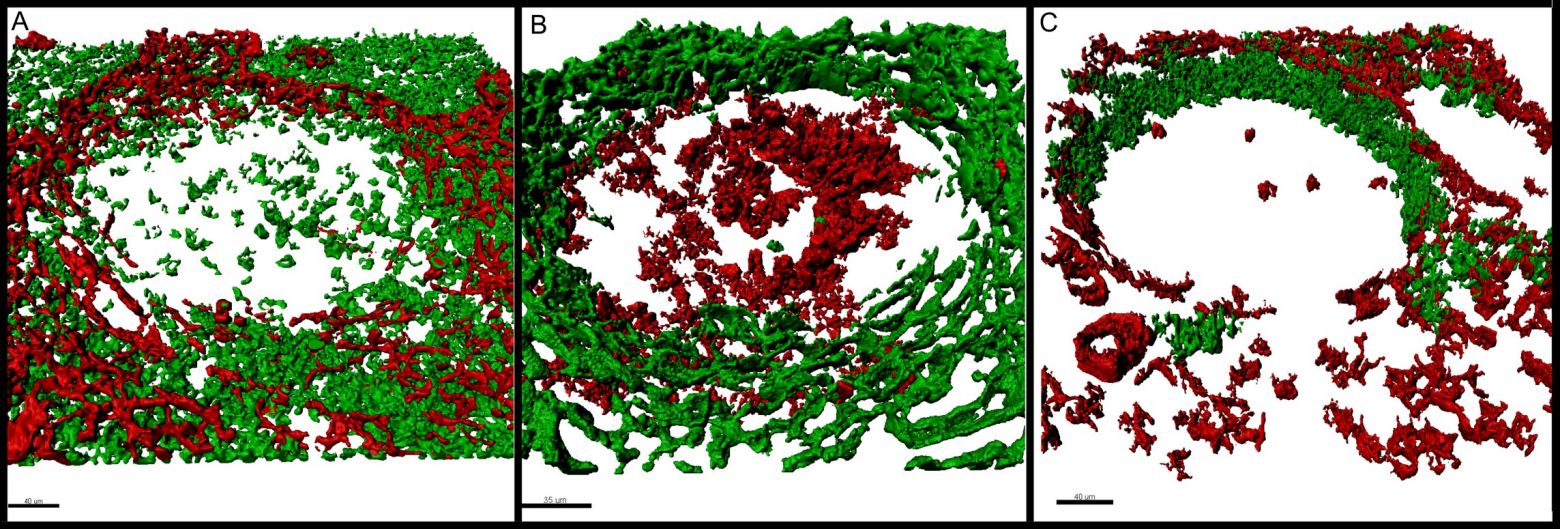
Description of the T-zone, the follicular mantle and the germinal centre in relation to the physical FRC boundary. (A) Alpha-actin^+^ (red) and CD3^+^ cells (green) relate to each other. The number of T-cells decreases abruptly towards the B-zone where the FRC wall is located. (B) Alpha-actin positive (green) and BCL6 positive (red) cells show proportion of walls towards the germinal centre. BCL6 positive germinal centre cells are situated within the physical boundary. Additionally, the BCL6 and alpha-actin negative surrounding between germinal centre and FRC wall indicates that the follicular mantle is within the compartmentalised area of the walls. (C) Alpha-actin-positive (red) and IgD positive (green) structures reveal orientation of FRC walls. The follicular mantle shows a polar orientation without or with only fragmented FRC walls towards the mantle zone. On the opposite pole, the germinal centre is compartmentalised by smaller FRC walls and is more permeable. The remaining germinal centre is enclosed from both sides by the FRC walls in form of a sufficient physical boundary. This staining combination shows properly that the orientation of the FRC walls shows a coherence with the follicular mantle. The maximum resolution of (A), (B) and (C) was set to 0.13 μm per pixel in the native microscopy dataset.

**Fig 4:**
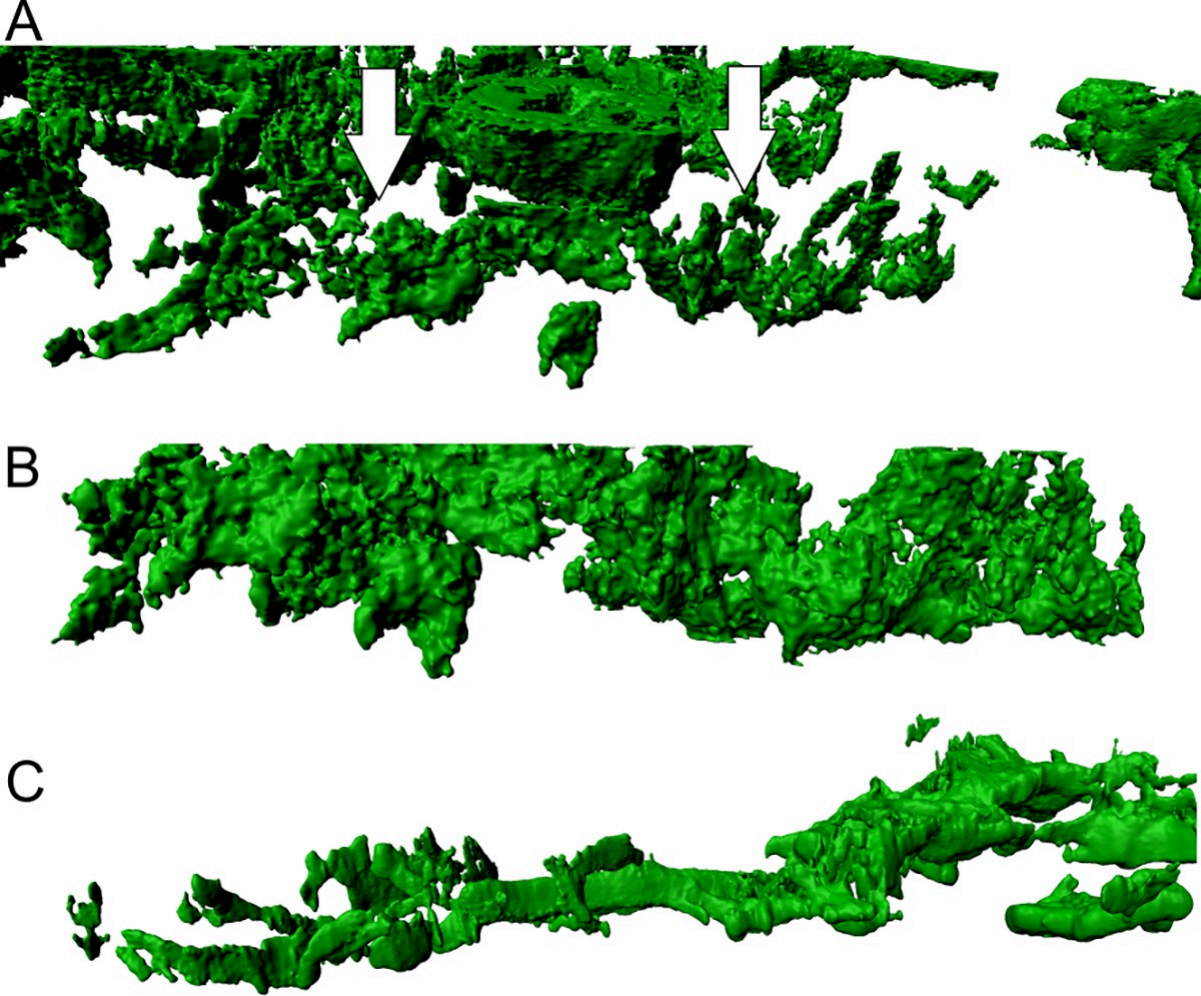
Morphological appearance of alpha-actin positive fibroblastic reticular cell (FRC) walls in angioimmunoblastic T-cell lymphoma (AITL). (A) Walls in AITL present themselves as less dense and not consistent as shown by the arrows (in white). The surface is inconsistent and permeable. (B and C) The FRC walls shown here display a rougher surface in comparison to LAD. In addition to that, they are more scattered and of variable morphology. The maximum resolution of (A), (B) and (C) was set to 0.13 μm per pixel in the native microscopy dataset.

We found alpha-actin expressing FRC walls (Fig 1) providing a defined compartmentalisation of the B-/T-zone. The wall structures in Fig 2 differed from the alveolar-like structures within the T-zone. FRC walls showed a clearly confined surface towards the germinal centre. These walls span over the whole thickness of the z-axis. 106 measurement points through the z-axis of FRC walls showed that 87 of them spanned at least two third over the specimen thickness. Cell communication is still possible through gaps within the walls. Furthermore, walls in LAD have shown a specific length in the circumcision of a germinal centre. Between unrelated walls, there is an opening that is not fragmented (Fig 2). These FRC boundaries as seen in Fig 3 feature a nearly consistent orientation. Towards the follicular mantle is a permeable zone without or with only fragmented physical barrier. The other pole is less permeable due to a smaller wall, whilst both sides between those areas show clear compartmentalisation through FRC walls. Tissue staining for BCL6 and IgD displays that the FRC walls compartmentalise GC including the follicular mantle. Further staining against CD3 has shown that the number of T-cells decreases within the B-zone behind the FRC wall. It is prominent that groups of T-cells accumulate on the side of the B-zone of the FRC boundary. The wall-like structures can vary in lymphomas like the AITL. To further specify the morphology in AITL (Fig 4), FRC wall structures have a less smooth surface and are more porous throughout the entire length. Also, there is a variable wall orientation in AITL due to the proliferation of CD23 positive FDCs.

### Length measurements of wall-like structures

**Fig 5:**
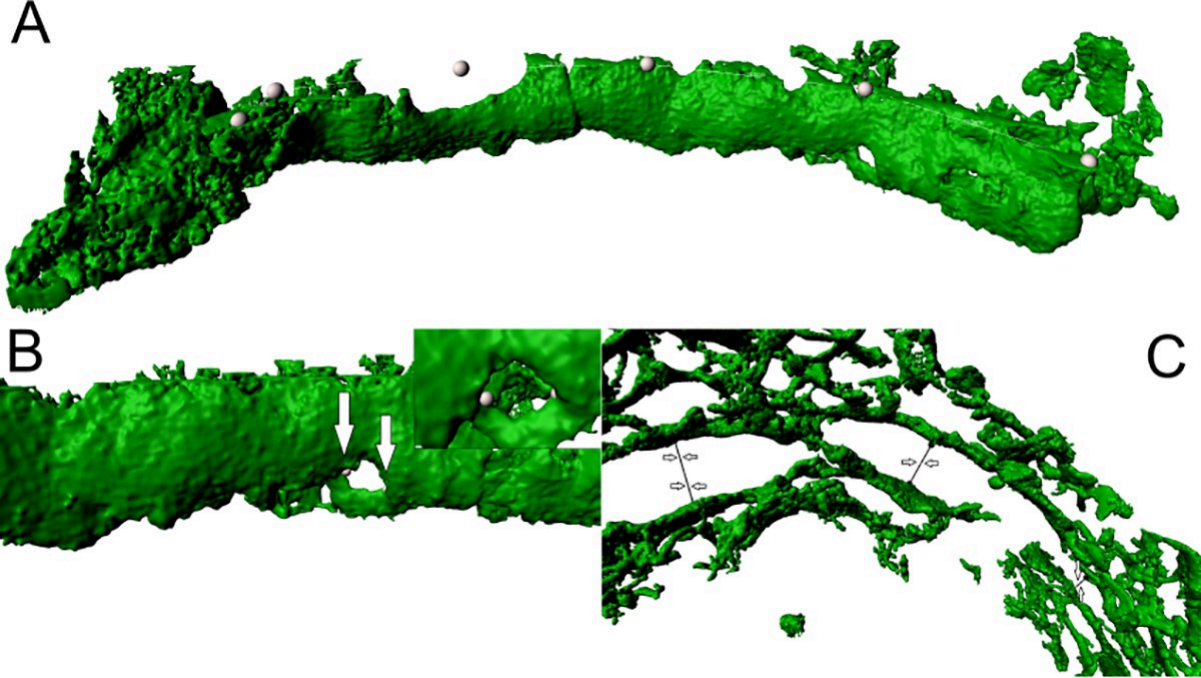
Measuring procedure. (A) FRC wall measurement in lymphadenitis (LAD) showing measurement points (white dots). The IMARIS measurement point application calculates wall length. (B) Alpha-actin positive FRC wall in LAD with a gap. Arrows pointing at measurement points that measure the diameter. In the upper right corner, an enlarged section of the gap is shown. (C) The measurement points are indicated by arrows. They depict the measurement procedure of the width of perifollicular sinuses in LAD. The maximum resolution of (A), (B) and (C) was set to 0.13 μm per pixel in the native microscopy dataset.

**Fig 6:**
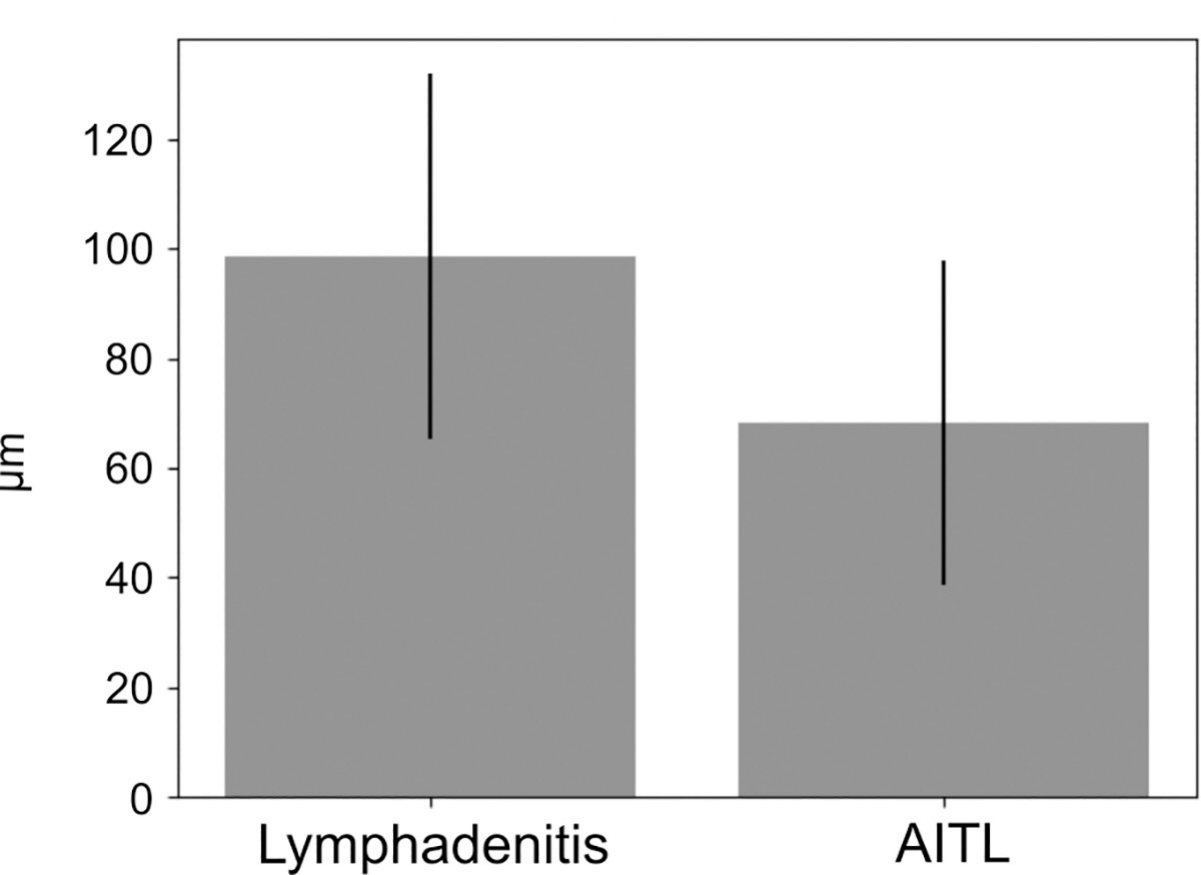
Boxplot of wall-length distribution. (A) Wall-lengths of fibroblastic reticular cells in cases of lymphadenitis (LAD) and angioimmunoblastic T-cell lymphoma (AITL) tend to show shorter walls in AITL (p<0.001).

The length of 78 FRC wall-like structures were measured in 14 images of LAD as in Fig 5. The AITL includes the amount of 56 FRC walls in 9 images. On average, we have found wall-like structures with a length of 98.65 μm (± 33.26) in cases of LAD and wall-like structures with a length of 68.24 μm (± 29.71) in cases of AITL. We revealed a significant difference of the wall-length distributions of p < 0.001 (p = 0.0000323676) by applying the Kolmogorov-Smirnov Test [19]. FRC walls in AITL are significantly shorter than in LAD, demonstrated in Fig 6. The thickness of FRC walls is 1.925 μm (± 0.56) in 42 FRC walls with 3 measurement points within each wall.

### Measurements of gaps within the FRC walls and perifollicular sinus

**Fig 7:**
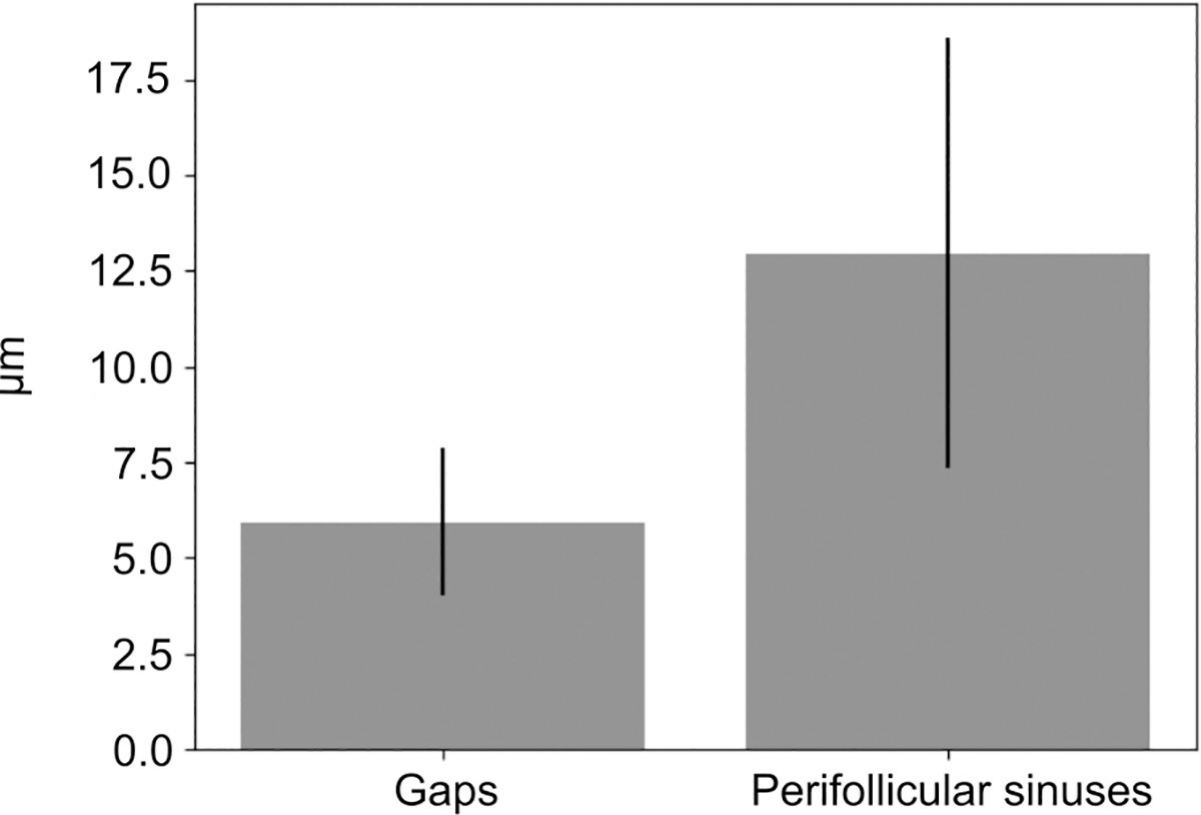
Analysis of alpha-actin positive structures. (A) Diameter of gaps within FRC walls & width of perifollicular sinuses. They appear to be separate structures complementing each other, rather than joining each other.

We measured the diameter of the gaps and the width of the perifollicular sinuses. On average, we quantified a diameter of gaps of 5.88 μm (± 1.965) in 11 images, including 61 structures. Considering the width of the perifollicular sinus, we measured on average 12.8 μm (± 5.59). We performed the measurements on 44 structures arising from 11 images. The results are visualized in Fig 7. The measurements were done in cases of LAD, considering the morphological difference and constitution in AITL FRC walls. AITL did not enable the examination due to the severe matrix destruction that severely affects the FRC walls.

## Discussion

The morphology of lymph nodes is usually investigated using 2 μm thin histological sections that provide a lot of information on different lymphoid and structural components forming lymph node compartments. Fibroblastic reticular cells providing a barrier between T-zone and B-cell area are well known [20]. Also an incline in FRC density towards the B-/T-border spanning 50 μm between the germinal centre and the T-zone has been described [2]. This border allowed only little B- and T-cell movement in between both zones [21]. However, structural components dividing compartments could not be investigated on thin sections because of technical reasons. There are major advances applying 3D laser scanning microscopy compared to 2D, looking at 2 μm thick conventional histological sections by light microscopy. For example, sinuses of the T-zone and FRC walls in the B-/T-border area with a complex morphology, which can be clearly visualized in 3D, cannot be illustrated in 2D. Laser scanning of thick sections offer a more detailed information about the reticular network showing volumes, details of surfaces, cellular and surface networks including walls, gaps and holes. It is known that the sinus borders influence the chemokine gradients guiding the different cell types along sinuses. In addition, many other morphological findings are of functional importance. For instance, alpha-actin positive walls turned out to be a mechanistic border between the T-cell area and the germinal centre, including the follicular mantle, forming a structure that hinders the B- and T-cell exchange in that area. Due to the orientation they still allow cell migration of various quantity of lymphocytes dependent on the localization within the B-/T-border. These walls however showed gaps and holes of an average of 5.88 μm (± 1.96 μm) in diameter that may be passed by B- and T- lymphocytes [22]. Our findings show T-cells that passed the boundary are gathering behind the FRC walls. This might indicate a functional importance in T-cell guidance for interaction with follicular B-cells. The perifollicular sinuses were wider than normal T-zone sinus structures, which were 50 μm apart from the B-/T-border. In contrast, in malignant lymphoma of AITL type, FRC walls showed a dramatic difference, more precisely, the separation of B- and T-cells was incomplete by large holes and gaps within these wall structures. A consequence of incomplete borders will be a dysregulation of the germinal centre’s cell reaction and an imbalance of homeostasis in and between B- and T-zones. The thickness and especially the variation of the wall length, as well as the details of the sinus structures can indicate the AITL due to changes in lymphatic connective tissue. In respect of the wall-length, we can distinguish between the functionality and the destruction of germinal centres. These 3D structures are on the one hand the consequence, on the other hand the cause of different niches for the lymphoid cells in reactive and neoplastic conditions. Modulations of cell speed are regulated by changing the surface structures and width of the sinuses and their shapes. Finally, 3D analysis of the germinal centre border should enable to differentiate between reactive and neoplastic lymph nodes. In neoplastic conditions, the form and degree of border destruction may give important information about the biology and the type of the malignant lymphoma. The severe destruction of germinal centre walls has been demonstrated in this paper in AITL, which is known to be a T-cell lymphoma showing a severe dysregulation of B- and T-cells. These patients typically develop common symptoms like fever, dysproteinaemia and infections that are difficult to cure, partly because of the abnormal compartmentalisation of the lymph node structure.

## Conclusion

The compartmentalisation of lymph nodes enables to control B- and T-cell immune reactions. Of special interest in this context is the 3D structure on the border between germinal centre and T-zone. We found a mechanical barrier in form of a wall that had a special length, thickness and permeability because of gaps and holes. In case of a malignant lymphoma of AITL type, the B- and T-cell border was partly broken down to enable to provide a severe B- and T-cell exchange that results in well-known serious immune dysregulation with all consequences these patients show clinically. Therefore, the complex morphology of T-zone reticular networks, especially within the B-/T-border region, are a result of immunological homeostatic or dysregulated immune reaction.

## Supporting information

S1 Table Data on measured wall length of lymphadenitis and angioimmunoblastic T-cell lymphoma. (XLSX)

S2 Table Data on gaps and perifollicular sinuses in lymphadenitis. (XLSX)
